# An Experience of Qualified Preventive Screening: Shiraz Smart Screening Software

**Published:** 2015-01

**Authors:** Parisa Islami Parkoohi, Hashem Zare, Gholamreza Abdollahifard

**Affiliations:** Department of Community Medicine, School of Medicine, Shiraz University of Medical Sciences, Shiraz, Iran

**Keywords:** Cancer, Chronic disease, Software, Quality improvement

## Abstract

**Background:**

Computerized preventive screening software is a cost effective intervention tool to address non-communicable chronic diseases. Shiraz Smart Screening Software (SSSS) was developed as an innovative tool for qualified screening. It allows simultaneous smart screening of several high-burden chronic diseases and supports reminder notification functionality. The extent in which SSSS affects screening quality is also described.

**Methods:**

Following software development, preventive screening and annual health examinations of 261 school staff (Medical School of Shiraz, Iran) was carried out in a software-assisted manner. To evaluate the quality of the software-assisted screening, we used quasi-experimental study design and determined coverage, irregular attendance and inappropriateness proportions in relation with the manual and software-assisted screening as well as the corresponding number of requested tests.

**Results:**

In manual screening method, 27% of employees were covered (with 94% irregular attendance) while by software-assisted screening, the coverage proportion was 79% (attendance status will clear after the specified time). The frequency of inappropriate screening test requests, before the software implementation, was 41.37% for fasting plasma glucose, 41.37% for lipid profile, 0.84% for occult blood, 0.19% for flexible sigmoidoscopy/colonoscopy, 35.29% for Pap smear, 19.20% for mammography and 11.2% for prostate specific antigen. All of the above were corrected by the software application. In total, 366 manual screening and 334 software-assisted screening tests were requested.

**Conclusion:**

SSSS is an innovative tool to improve the quality of preventive screening plans in terms of increased screening coverage, reduction in inappropriateness and the total number of requested tests.

## Introduction

Nowadays, the world is faced with the epidemics of non-communicable chronic diseases (NCDs). Such diseases are the leading cause of morbidity and global mortality. NCDs, while largely preventable, impose tremendous human, social, and economic costs on communities. In this context, proper preventive screening is an effective form of intervention.^1^ Preventive screening is a cost-effective and high-quality phase of health care in which a non-apparent risk factor or disease is identified by the application of laboratory tests, physical examinations or other procedures.^2^^,^^3^

In our location (Shiraz Medical School, Shiraz, Iran), the school’s occupational health and safety officer instructed manual periodic health examinations and preventive screening for 330 non-academic staffs from the year 2009. Following initial review and monitoring phase, the assigned medical team identified several shortcomings with respect to the individual’s attendance and requisition of screening tests. Considering the importance of screening in tackling high-burden NCDs, it was decided to document and correct identified flaws in order to improve the screening quality. The key task, in our opinion, was to reduce the total number of the requested tests by eliminating needless requests and solely focus on essential requests where regular attendance in terms of convenience could be motivated.

The motivation to resort to a software-assisted procedure to improve screening quality stem from successful experiments carried out using screening tools such as “Family Healthware”, “Child Health Improvement through Computer Automation (CHICA)” or “Palm Prevention”. Family Healthware is developed by the “Centers for Disease Control and Prevention (CDC)” and is a family-history screening tool to prevent common chronic diseases. CHICA is a targeted screening system for iron deficiency anemia and tuberculosis in children. Palm Prevention is a free software tool for Palm operating system personal digital assistants (PDAs) that provides quick access to preventive guidelines in a patient-specific manner at the point of care and improves adherence to five preventive measures in primary care.^4^^-^^6^

Subsequently, in collaboration with an information technology team, dedicated software tool called “Shiraz Smart Screening Software” (SSSS) was developed. This tool was then used in 2012 for preventive screening of non-academic staffs in a software-assisted manner. The underlying philosophy in this study was to determine the extent by which the SSSS tool could improve screening attendance and how it would positively influence the total number of appropriate requested tests. 

## Materials and Methods

Periodic health examination and screening of the staff was carried out in a health clinic during 2009-2011 by the occupational health and safety affairs team affiliated with the Shiraz Medical School. The team included two general physicians and two occupational health practitioners, under the supervision of the Department of Community Medicine. Information such as a brief history, physical examination, and screening test requisition were registered by the team in the form of paper-based health records. 

Our team, while monitoring the above process and after analyzing the registered health records, identified several predominant shortcomings in terms of irregular-time attendance and considerable overuse/underuse of the screening tests. Subsequently, it was decided to introduce software-assisted approach (SSSS) to correct for the shortcomings by a simultaneous intelligent computerized screening of several high-burden chronic diseases. It is worth mentioning that the general physicians identified additional issues such as time limits, difficulty in real-time decision-making on the requisition of screening tests for several diseases as well as the inaccessibility to convenient preventive guidelines.

During the initial phase of software development, the following diseases and conditions for screening were considered: diabetes mellitus, hypertension, hyperlipidemia, coronary artery diseases, metabolic syndrome and cancer of the skin, colorectal, prostate, breast and cervix. Selection criteria were high burden and public health concerns, clear definition, known risk factors, and availability of effective all-level preventive services in the society. For a better evaluation of the first release of the software, only a limited number of diseases were considered. 

In the next step, screening processes workflow was described in the form of a Request for Proposal (RFP). This was drawn up in accordance with the available evidence-based preventive guidelines and local considerations to ensure appropriateness of the screening tests.^7^^-^^9^ Additionally, the provision of general and disease-specific health recommendations as well as electronic reminder was included. The reasoning behind the inclusion of the reminder notification was incorporated and explained in the REP document. It described evidence-based advantages of reminders to increase screening uptake and quality reports of computerized reminder systems such as a Mammography Fast Track program for identifying and contacting patients overdue for screening.^10^^-^^12^

In addition, the following essential concepts were considered:

- Immediate and accurate identification of high risk persons and conditions
- Client’s “no need to visit” to check the completion of screening tests and referrals
- Access to the most recent profile of individuals at all times and places
- Continuity of screening despite a change of service provider
- Confidentiality and security of client information
- Provide screening-related data processing, presented in a user friendly screening- specific interface
- Software flexibility to accommodate guideline alterations
- Availability of statistical report
- Compatible with Windows and web-based systems

The final REP reports were reviewed by two community medicine specialists and then passed on to the information technology team for software implementation. Software interface was designed based on reviewing the concept in order to ensure consistency with RFP and best clinical practice. In addition, user friendliness and ease of data entry were considered by utilizing itemized check-box or combo-box interfaces. The steps that a user would perform screening through dialog boxes are described below. 

*Identification Data Registry*

As part of the Initial phase of the screening, personal information such as national identification code, name, surname, date of birth, gender, marital status and other details are entered. The system assumes the national identification code as a client-specific identification code.

*Medical History Registry*

Relevant screening data on client’s past medical history, including known diseases, cancer, and familial and smoking history are registered. 

*Physical Examination Registry*

Data related to the measured blood pressure (with intelligent severity categorization), height, weight and waist circumference (with automated identification of abdominal obesity) are registered. The Body Mass Index (BMI) is then automatically calculated, categorized and displayed. In addition, findings from other physical examinations are registered and displayed via default items. 

*Screening Results Registry*

Values and the date of the quantitative screening test are registered. The incorporated textbox interface allows entering test reports such as Pap smear, mammography and colonoscopy.

*Intelligent Recommendation of Screening Plan*

This window displays the main task of the software. The system can automatically identify indicated screening tests and examinations plus the due date. In addition, intelligent detection and representation of conditions such as metabolic syndrome and other new diseases as well as the calculation of the Framingham Risk Score is done based on the registered data in the previous steps. Likewise, the software can introduce the type of specialists required for referrals and automatically displays general and screened disease-specific messages.

*Sending Reminder Notification*

Smart reminder is done by sending e-mail and short message services. The scheduling for sending a reminder is two weeks, one week and three days before the due date. In overdue cases, the system sends a reminder three days, one week, and two weeks after the due date.

At the end of the programming phase, a software demo version was released, evaluated, and verified by simulating virtual scenarios as well as quality control by academic specialists on each intended disease. To evaluate software usability, screening was done on 23 volunteers from different departments and their experience and feedback were noted. Following minor software modifications, the final version of the software was released and deployed as software-assisted screening.

Initially, as customary, the school staffs were called upon by our co-workers in the occupational health and safety affairs for annual health examination and screening at a health clinic during 2012. These health services were given during four months from June to September (the schedule was based on staff’s willingness) by the members of the community medicine research group. Attendees were debriefed on the software characteristics and steps to follow after which verbal consent was obtained. Personal identification data, screening-based history, and physical examinations were performed and the corresponding data as well as the available results from prior screening tests were registered in the system. Finally, volunteers were informed about the output of the software, including indicated screening tests with their due date as well as any required referral(s) to specialist(s) and related health advice. The time spent on each volunteer was 20 to 30 minutes.

It should be noted that the inclusion criterion of the study was employment at the school and the exclusion criterion was unwillingness to attend. In accordance with site regulation, approval from the Research Ethics Committee was obtained. 

Quasi-experimental study design was selected to assess the capability of the software-assisted screening for improving screening quality. The quality of previous manual screening and the current software-assisted screening was assessed by comparing the proportion of non-attendance and irregularly-timed attendance as well as the total number and the inappropriateness of the requested screening tests. 

Irregularly-timed attendance proportion was defined as the total number of individuals who participated in periodic health examination and screening (with intervals more than one year) divided by the total number of participants.

The measurement of the total number of tests was according to the count of the observed relevant results on paper-based health records (due to incompleteness of records) and the software output report for manual and software-assisted screening. For inappropriateness proportion calculation of each kind of screening test in manual screening, the total number of indicated, but not requested (underused) tests and non-indicated but requested (overused) tests was divided by the total number of requested tests. Since software programming was based on available competent preventive guidelines, the inappropriateness proportion in software-assisted screening was assumed zero. The comparisons were performed by descriptive analysis in SPSS version 15. 

## Results

Reviewing paper-based health records revealed that during 2009 to 2011, following the communal call, 24%, 21%, and 37% of the employees underwent manual periodic heath examination and screening respectively (27% on average).

During periodic health examination and screening by the software, following the initial phone invitation, out of 330 employees, 281 (85%) were willing to participate from which 261 (79%) attended and were screened by the software. The participants included 52% women and 48 % men with mean±SD age of 36.4±8.68 years (range: 23-62 years).

According to the existing paper-based health records, 146 (94%) of the attending individuals had non-attendance or irregularly-timed attendance prior to the software implementation. This proportion for software-assisted screening would be cleared after at least one year.

The corresponding screening test results in paper-based health records elucidated inappropriate screening, as shown in tables 1 and 2. It should be noted that, assuming 0% as the inappropriateness proportion in software-assisted screening, the difference in inappropriate screening tests between these procedures is statistically significant. The data on the requested tests in manual and software-assisted screening are shown in table 3.

**Table 1:** Status of inappropriate screening for non-gender-specific diseases among the employees of Shiraz Medical School, before the use of the screening software, (from 2009 to 2011)

**Screening test**	**Proportion of non-requested tests in indicated cases**	**Proportion of requested cases in non- indicated cases**	**Inappropriate tests in a total of 261 cases **
Fasting plasma glucose	59/167 (35.32%)	49/94 (52.12%)	108 (41.37%)
Lipid profile	49/145 (33.79%)	59/116 (50.86%)	108 (41.37%)
Occult blood	22/23 (95.65%)	0/238 (0%)	22 (0.84%)
Flexible sigmoidoscopy or colonoscopy	5/5 (100%)	0/256 (0%)	5 (0.19%)

**Table 2:** Status of inappropriate screening for gender-specific diseases among the employees of Shiraz Medical School, before the use of the screening software, (from 2009 to 2011)

**Screening test**	**Proportion of non-requested tests in indicated cases**	**Proportion of requested cases in non-indicated cases**	**Proportion of inappropriate tests in total cases **
Mammography	24/44 (54.54%)	2/92 (2.17%)	26/136 (19.9%)
Pap smear	48/78 (61.53%)	0/58 (0%)	48/136 (35.29%)
Prostate specific antigen	13/13 (100%)	1/112 (0.9%)	14/125 (11.2%)

**Table 3:** Frequency of requested screening tests for employees of Shiraz Medical School by manual (2009 to 2011) and software-assisted screening (2012)

**Screening test**	**Number of requested tests by manual screening**	**Number of requested tests by software-assisted screening**
Fasting plasma glucose	157	156
Lipid profile	155	85
Occult blood	1	12
Flexible sigmoidoscopy or colonoscopy	0	5
Pap smear	30	40
Mammography	22	26
Prostate specific antigen	1	10
Total	366	334

## Discussion

Shiraz Smart Screening Software (SSSS) is an innovative tool for quality improvement in preventive screening health services. This smart screening-specific software is applicable for periodic health examination, simultaneous preventive screening of multiple chronic diseases, and sending reminders.

Our results indicate that, a substantial percentage of volunteers had no regularly-timed periodic health examinations prior to software implementation. In a systematic review, conducting such examinations in clinics was verified due to its effective delivery of preventive services and addressing the health concerns of clients.^13^ It appears that the SSSS tool has been successful in increasing the number of attendance due its convenience and time efficiency.

Prior to screening by SSSS, there were considerable numbers of overused or underused screening tests. This would be more prominent if one considers that even reflecting on the requests for prior non-requested tests; the number of requested tests with the software has been reduced. This shortcoming could be due to disregard for the existing risk factors, unavailability, or incorrect usage of guidelines, etc.

Despite exposure to numerous preventive guidelines, physicians have a challenge in applying such guidelines in clinical practice.^6^ A national survey in the USA revealed that, on average, only 19.5% of primary care physicians requested various colorectal cancer screening tests in a manner consistent with the available guidelines. Physicians working on electronic health records, did better (OR=2.31) than those with paper-based health records.^14^

We considered consistency with the available competent preventive guidelines in all steps of programming, as well as implementation and usability testing of the software to offer appropriate screening test recommendations.

The total frequency of requested screening tests has been decreased by the software-assisted screening. However, despite the fact that, due to practical reasons, a fewer number of requested tests is favorable, the difference between the number of manual and software-assisted fasting plasma glucose tests was minimal. The software correctly identified individuals with BMI>25 and other relevant risk factors, which was not the case in the manual process. Furthermore, the increased number of post software-assisted screening in colorectal, prostate, breast and cervical cancer cases are due to the request of truly indicated cases, something that was not considered in manual screening.

In preventive services, identification of the screening-eligible clients and direction of clinical data are the least expected functionality of an information technology system.^12^^,^^15^ The software presented in this report, in addition to screening for cancer, is capable of intelligent identification of conditions such as high normal hypertension, borderline hyperlipidemia and impaired fasting glucose with scheduling for follow up. This is a valuable functionality for health maintenance of individuals.

Participation of target groups is one of the most important influencing factors on the efficiency and effectiveness of screening programs.^3^ Brouwers and colleagues reviewed and suggested some interventions for increasing the uptake of cancer screening especially for breast, cervix and colorectal cancers (client reminders were optional).^16^ The SSSS tool has reminder notification capability via short message service and e-mail. However, the result from this functionality could not be presented due to its recent implementation (under one year). Note that the first reminder for the next health examinations and follow up of screening tests is set after the first year.

Our study has some limitations in terms of not leveraging on the opinion and experience of physicians who deliver community-wide preventive services and their clients, before or during software development. However, considering inadequate delivery of preventive services in our community health system, due to the dominance of treatment-centered views, this limitation is justifiable. Another limitation relates to the low number of participants, which is inadequate for overall validity. This is mainly because the software is recently released, but the numbers would increase following immediate market promotion and initial success reports. Despite the above-mentioned limitations, the SSSS software has been successfully executed in a user-friendly manner. A specific advantage of the software is simultaneous user assistance for five types of site-specific cancers as well as other chronic diseases in 20 to 30 minutes. The time saving benefit of this capability is evident.

In a study by Yarnell and colleagues, it was shown that problems such as increased number of clients, large number of preventive recommendations and providing care for urgent cases, restrict physicians to deliver all of the client-targeted preventive services, even without considering the spent times for reviewing the records.^17^ On the other hand, it was shown that combined screening plans are more effective, feasible, and popular than individual screening plans.^18^ The SSSS software offers this service and the screened individuals expressed their satisfaction during the study.

For a better evaluation of the software (such as validity and reliability) and its impact on increasing the screening delivery and uptake, long-term follow up of the screened participants as well as execution of software on larger and different populations, settings and users is necessary. Moreover, documented investigation regarding its time saving feature and cost analysis is recommended. 

## Conclusion

In line with a strategy for reducing chronic diseases, Shiraz Smart Screening Software (SSSS) is an innovative tool for quality improvement of the preventive screening plans through increased screening coverage, decreased inappropriateness as well as the total number of requested tests. Pending further studies, it can be deployed extensively in a time and cost saving manner. 
